# Evaluation of Red Cell Distribution Width to Lymphocyte Ratio as Potential Biomarker for Detection of Colorectal Cancer

**DOI:** 10.1155/2019/9852782

**Published:** 2019-07-31

**Authors:** Jiahao Huang, Yang Zhao, Lin Liao, Shun Liu, Shaolong Lu, Changtao Wu, Chuanyi Wei, Shaoqiang Xu, Huage Zhong, Junjie Liu, Yun Guo, Sen Zhang, Feng Gao, Weizhong Tang

**Affiliations:** ^1^Department of Gastrointestinal Surgery, Guangxi Clinical Research Center for Colorectal Cancer, Affiliated Tumor Hospital, Guangxi Medical University, Nanning, Guangxi, China; ^2^Department of Colorectal and Anal Surgery, The First Affiliated Hospital, Guangxi Medical University, Nanning, Guangxi, China; ^3^Department of Radiology, Affiliated Cancer Hospital, Guangxi Medical University, Nanning, Guangxi, China; ^4^Department of Clinical Laboratory, The First Affiliated Hospital, Guangxi Medical University, Nanning, Guangxi, China; ^5^Department of Epidemiology, School of Public Health, Guangxi Medical University, Nanning, Guangxi, China; ^6^Department of Hepatobiliary Surgery, Affiliated Cancer Hospital, Guangxi Medical University, Nanning, Guangxi, China; ^7^Department of General Surgery, Red Cross Hospital of Yulin City, Yulin, Guangxi, China; ^8^Department of Ultrasound, Affiliated Tumor Hospital, Guangxi Medical University, Nanning, Guangxi, China

## Abstract

**Background and Aim:**

Colorectal cancer (CRC) is the third most lethal cancer globally. This study sought to determine the feasibility of using red cell distribution width-to-lymphocyte ratio (RLR) as a tool to facilitate CRC detection.

**Methods:**

Seventy-eight healthy controls, 162 patients diagnosed with CRC, and 94 patients with colorectal polyps (CP) from June 2017 to October 2018 were retrospectively reviewed. Clinical data were obtained to analyze preoperative RLR level, and receiver operating characteristic (ROC) curve analysis was performed to estimate the potential role of RLR as a CRC biomarker.

**Results:**

RLR was higher in patients with CRC than in healthy participants (*P* < 0.05). ROC analysis indicated that combined detection of RLR and CEA appears to be a more effective marker to distinguish among controls, CP, and CRC patients, yielding 56% sensitivity and 90% specificity. RLR levels were significantly greater in those who had more advanced TNM stages (*P *< 0.05) and patients with distant metastasis stages (*P *< 0.05).

**Conclusions:**

RLR might serve as a potential biomarker for CRC diagnosis.

## 1. Introduction

Colorectal cancer (CRC) is among the most frequently detected types of cancer and is third most deadly cancer with worldwide [1]. In 2014 in China alone, there were 370,000 new cases and 180,000 deaths attributable to CRC [2]. Screening efforts can help to prevent deaths associated with CRC. Despite the fact that stool-based tests, colonoscopy, and carcinoembryonic antigen (CEA) tests or a combination assay of CEA and carbohydrate antigen 19-9 (CA19-9) are recommended, they often fail to detect early disease. Therefore, it is urgent to identify new, accurate, and sensitive indicators that can detect CRC and predict its prognosis.

Red blood cell distribution width (RDW) reflects RBC size heterogeneity. It is routinely calculated on complete blood count analyzers. For several decades, RDW was used in clinical settings only to test for anemia or related maladies. Recently, however, many studies have revealed that RDW may be used to predict the severity of inflammatory bowel disease (IBD) irrespective of anemia [3, 4]. CRC has long been known to be linked with a chronic inflammatory state, and this can be present even when tumor development is in the initial stage. Therefore, the clinical significance of RDW in CRC diagnosis and prognosis has attracted much attention [5, 6]. RDW-to-lymphocyte ratio (RLR), a combination of the two parameters, is an easily-acquired parameter using blood routine tests. We believe that no study to date has studied the diagnostic role of RLR in patients with CRC; RLR might be more powerful than one parameter alone for diagnosis of CRC.

In the present study, we assessed RLR levels in CRC patients and in individuals with colorectal polyp (CP) as well as in normal controls. The diagnostic value of RLR in CRC was explored in detail.

## 2. Materials and Methods

### 2.1. Patient Characteristics

We retrospectively analyzed the medical data from patients newly diagnosed with CRC at the first affiliated Hospital of Guangxi Medical University (Nanning, China) from June 2017 to October 2018. Patients with CP and healthy individuals both served as age-matched control groups. Individuals were excluded from the study if they had hematological disorders, kidney disease, acute/chronic infections, coronary artery disease, hypertension, diabetes mellitus, and medical treatment with anticoagulant, if they had undergone transfusions in the previous 3 months, if they had received neoadjuvant therapy, or if they had other cancers. Those who had adenocarcinomas confirmed histologically were enrolled in the CRC study group. The institutional medical ethics review board of the first affiliated Hospital of Guangxi Medical University approved this study, and all patients gave written informed consent.

### 2.2. RLR, CEA, and CA19-9 Measurements

We extracted laboratory data of all subjects on admission, including hematological parameters as well as measured levels of tumor markers. RDW and absolute lymphocyte counts (L) were determined using a Coulter LH780 Hematology Analyzer (Beckman Coulter Inc., Fullerton, CA, USA). The RLR level was calculated using the following ratio: RDW%/lymphocyte count. Serum CEA and CA19-9 were measured on a Cobas e601 Analyzer (Roche Diagnostics, Mannheim, Germany) based on manufacturer's instructions. The normal CEA cutoff level was 5 ng/mL, and for CA19-9 it was 37 U/mL.

### 2.3. Statistical Analysis

SPSS software (SPSS 25.0, Chicago, Illinois, USA) or GraphPad Prism version 7.0 (La Jolla, CA 92037 USA) was employed for all analyses. Continuous variables were expressed as means ± standard error of mean (SEM). Receiver operating characteristic (ROC) curves were employed to assess the diagnostic values and ideal cut-offs were determined using the Youden index [7]. The diagnostic accuracy was determined by area under the curve (AUC), and Delong's test was performed to compare the AUCs across different models [8]. Student's t-tests and one-way ANOVAs with Student-Newman-Keuls post hoc tests were used to compare data in this study as appropriate, with* P* < 0.05 considered statistically significant (two-tailed).

## 3. Results

### 3.1. RLR Levels in CRC Patients

**Figure 1:**
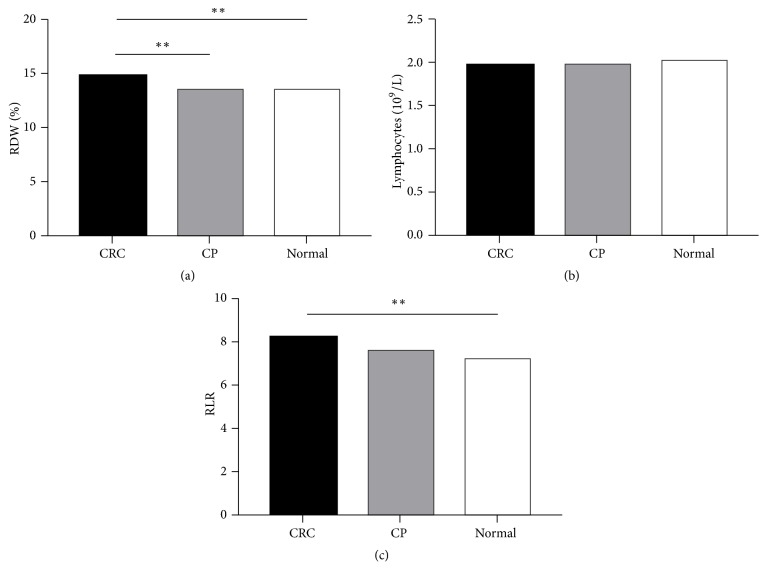
The levels of RDW (a), L (b), and RLR (c) were determined by hematology analyzer in CRC patients (N = 162), CP patients (N = 92), and healthy controls (N = 78). Data are presented as means ± SEM. *∗∗P* < 0.05.

We enrolled a total of 162 CRC patients, 92 CP patients, and 78 healthy participants from June 2017 to October 2018. Demographic characteristics, RDW, lymphocyte count, and RLR level are shown in Table 1. Age and gender were comparable between groups. Our data demonstrated that the level of RDW in CRC patients was significantly higher than those of CP patients and healthy participants (*P* < 0.05). More importantly, RLR levels were significantly higher in CRC patients than in healthy participants, while no significant differences were found between CRC patients and CP patients (Figure 1,* P* < 0.05).

### 3.2. Assessment of RLR as a CRC Diagnostic Biomarker

We assessed the potential for RLR to serve as a diagnostic CRC biomarker as compared to CEA and CA19-9. An ROC curve was generated to estimate the diagnostic ability of RLR for the diagnosis of CRC patients or controls. CRC patients had significantly higher CEA levels than did CP patients and healthy volunteers; however, this phenomenon was not observed with CA19-9. Furthermore, the ROC area under the curve (AUC) for CA19-9 suggested it might not be a useful marker for CRC clinical diagnosis (*P* > 0.05).

**Figure 2:**
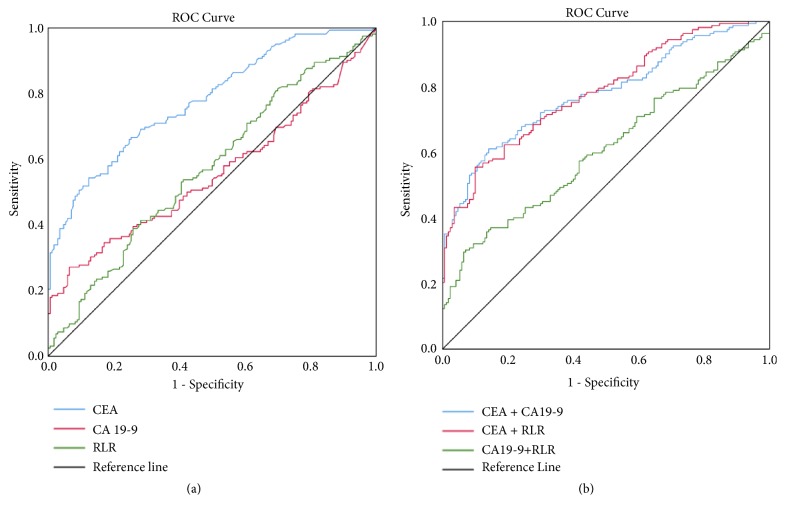
Receiver operating characteristics (ROC) curve analysis of the diagnostic performance of RLR in comparison to CEA and CA19-9. (a) ROC curves of RLR, CEA, and CA19-9 alone for discriminating CRC patients. (b) ROC curves of CEA + RLR, CA19-9 + RLR, and CEA + CA19-9 for discriminating CRC patients.

ROC analysis suggested that 8.21 was the optimal RLR cutoff (AUC: 0.571, sensitivity: 41%, specificity: 72%; Figure 2(a)), and 5 was the CEA cutoff (AUC: 0.779, sensitivity: 37%, specificity: 97%). However, diagnostic performance did not improve when RLR was compared with CEA (Figure 2(b),* P* > 0.05). Therefore, RLR in conjunction with CEA was further analyzed for combined detection. As shown in Table 2, the AUC of the combined detection of RLR and CEA was not significantly greater than the combination of CEA and CA19-9. Importantly, when RLR and CEA were combined, sensitivity and specificity were 56% and 90%, respectively.

These findings suggest that combined detection of RLR and CEA performed better than detection of CEA alone for identifying CRC patients, with higher sensitivity. In addition, diagnostic specificity was greater when RLR and CEA were combined for CRC detection than with CEA plus CA19-9.

### 3.3. The Relationship between RLR and CRC Stage and Metastasis

As shown in Table 3, RLR levels were substantially increased in CRC patients with more advanced TNM staging (*P* < 0.05). Further analysis revealed that RLR was linked to distant metastasis stage (*P* < 0.05). Nevertheless, we detected no significant difference between RLR level and location, primary tumor stages, lymph node stages, differentiation, and tumor size. Taken together, these data suggest that RLR correlated with clinicopathological characteristics in individuals who have CRC.

## 4. Discussion

CRC is major cause of death, with a 50% 5-year survival and 10-year survival > 20% [9–12]. Moreover, CRC is usually asymptomatic until late in its course, resulting in a quarter of patients initially presenting with metastases in the lungs or liver [13]. CRC can develop from precancerous polyps over an average of 10+ years [14, 15]. Histopathology can distinguish between polyps that are adenomas and those that are hyperplastic. Importantly, several lines of evidence suggested that both adenomas and some subtypes of hyperplastic polyps were precursor lesions for CRC origin [16, 17]. Colonoscopy is an effective means of preventing CRC via early detection and removal of problematic lesions; however, it is expensive and is associated with risks of perforation as well as risks associated with sedation and aspiration. Therefore, noninvasive, accurate, and sensitive diagnostic strategies are essential when there is a suspicion of CRC.

Some studies suggest that prolonged inflammation in the intestine is related to CRC, and this is likely to influence CRC initiation and development [18]. Previous work suggested that the Glasgow prognostic score, neutrophil-to-lymphocyte ratio, platelet-to-lymphocyte ratio, and lymphocyte-to-monocyte ratio are parameters related to inflammation that might be associated with CRC-specific survival [19]. High values of RDW imply greater variation in the distribution of RBC volume in circulation. Earlier reports suggested that elevated RDW values were linked to more advanced stages of CRC. Moreover, RDW was found to be relevant to metastasis of CRC [6]. Lymphocytes serve essential roles in the production of cytokines and the cytotoxic killing of cells, thereby preventing tumor growth/metastasis [20, 21]. Therefore, RLR, the combination of these two parameters may reflect a balance between tumor inflammation and antitumor immunity.

We assessed the RLR levels of 162 CRC patients and found these levels were higher in CRC patients than in healthy volunteers. To our knowledge, we are the first to report that RLR can effectively differentiate CRC patients from CP patients and healthy participants. We also assessed how well RLR performed as a diagnostic marker of CRC using ROC analysis and found that RLR plays a vital role in the diagnosis of CRC, with a 8.21 cut-off value, 41% sensitivity, and 72% specificity. Previously, levels of CA19-9 and CEA were commonly used as tumor markers useful for determining CRC staging [22]. These two markers are relatively insensitive, however, limiting their use when screening for CRC. It has been widely recognized that marker combinations have better diagnostic performance than do individual markers. Nevertheless, our data do not support use of CA19-9 when screening for or monitoring CRC patients. Therefore, the combined detection of RLR and CEA can achieve better diagnostic performance than can CEA in conjunction with CA19-9 for identifying CRC patients, with improved specificity.

The underlying mechanisms pertaining to the relevance of RLR in the context of CRC remain to be determined. Recent work suggests that higher RLR levels correspond to increased RDW numbers and/or lower lymphocyte levels. It is known that the pathogenesis of CRC is closely related to ongoing inflammation. Because inflammation positively correlates with RDW [23–25], ongoing intratumoral inflammation results in an elevation of RDW. Alternatively, patients with CRC are prone to hemorrhaging, and this impairs iron storage, resulting in variations in RBC size and increased RDW.

Lymphocytes are important components of white blood cells, playing crucial roles by driving antitumor immunity and tumor cell apoptosis, thereby improving CRC patient survival [26, 27]. A decreased number of lymphocytes could impede antitumor immune responses and further facilitate tumor metastatic potential [26]. Evidence from several studies also suggested that low-tumor-infiltrating lymphocytes are significantly associated with poor survival in CRC [28, 29]. A metric that assessed both RDW and lymphocytes (such as RLR) has the potential for widespread clinical use as it can simultaneously reflect both of these processes.

Furthermore, we primarily focused on the association between RLR and clinicopathological characteristics of CRC patients. We found that RLR was most frequently elevated in advanced TNM stages and in CRC patients with distant metastases. Therefore, according to our data, greater RLR may indicate more robust inflammatory responses in response to more aggressive tumors and higher tumor burdens. Given the importance of TNM stage and distant metastasis in predicting CRC patient outcomes, our finding suggests that RLR might function as a prognostic CRC biomarker.

We acknowledge that there are several limitations of the present study. This was a retrospective single-center study. Second, lacking follow-up data made it challenging to fully appreciate the relevance of RLR in evaluating the overall survival in CRC patients.

In conclusion, the present study suggests that RLR might serve as a potential biomarker for CRC, predicting its progression. In clinical practice, combined detection of RLR and CEA should be evaluated as possible effective markers for CRC diagnosis, despite the fact that additional studies are needed to validate our findings.

## Figures and Tables

**Table 1:** Comparisons of laboratory parameters among the patients of colorectal cancer, colorectal polyp, and healthy controls.

Parameter	Colorectal cancer	Colorectal polyp	Healthy controls
	N = 162	N = 92	N = 78
Age, years	53.99 ± 11.44	52.67 ± 12.06	52.9 ± 7.41
Gender (M/F)	97/65	58/34	51/27
WBC, ×10^9^/L	6.76 ± 1.68*∗*	6.25 ± 1.5	6.42 ± 1.6
Hb, g/L	119.62 ± 23.81*∗*	134.1 ± 16.09	146.25 ± 15.05*∗∗*
CEA, U/mL	13.9 ± 38.73*∗*	2.11 ± 1.66*∗∗∗*	1.32 ± 1.12*∗∗*
CA19-9,U/mL	203.97 ± 1270.05	8.79 ± 5.94	14.63 ± 9.76
Red cell distribution width (RDW), %	0.15 ± 0.03*∗*	0.14 ± 0.01	0.14 ± 0.01*∗∗*
Lymphocytes, ×10^9^/L	1.97 ± 0.57	1.98 ± 0.61	2.03 ± 0.57
RDW to lymphocyte ratio (RLR)	8.21 ± 3.45	7.59 ± 2.88	7.2 ± 2.05*∗∗*

*∗P*<0.05 between CRC and CP.

*∗∗P*<0.05 between CRC and healthy controls.

*∗∗∗P*<0.05 between CP and healthy controls.

**Table 2:** Diagnostic value of RLR, CEA, and CA199 alone and combined biomarkers for distinguishing CRC patients from colorectal polyp patients and healthy participants.

Variables	AUC	Cut off	Sensitivity	Specificity	95% confidence interval	
					upper limit	lower limit
RDW to lymphocyte ratio (RLR)	0.571	8.21	41%	72%	0.730	0.828
CEA	0.779	5.00	37%	97%	0.493	0.619
CA19-9	0.556	37.00	16%	99%	0.509	0.632
CEA + CA19-9	0.779		61%	86%	0.729	0.829
CEA + RLR	0.782		56%	90%	0.734	0.831
CA19-9+RLR	0.607		30%	93%	0.545	0.668

**Table 3:** Relationship between RLR and pathological characteristics in CRC patients.

Variables	N	RLR	*P* value
Gender			
Male	97	8.06 ± 3.00	0.489
Female	65	8.40 ± 4.05	
Location			
Right-sided colon cancer	33	9.01 ± 3.31	0.138
Left-sided colorectal cancer	129	8.01 ± 3.47	
TNM stage			
I+II	75	7.57 ± 3.45	*0.028*
III+IV	87	8.76 ± 3.38	
pT stage			
T1	4	6.94 ± 2.22	0.314
T2	28	8.74 ± 4.89	
T3	60	8.63 ± 3.21	
T4	70	7.71 ± 2.97	
pN stage			
N0	79	7.79 ± 3.72	0.131
N1+N2	83	8.61 ± 3.14	
pM stage			
M0	147	8.04 ± 3.38	*0.048*
M1	15	9.88 ± 3.83	
Differentiation			
Poor	18	9.19 ± 4.58	0.278
Moderate	121	7.96 ± 2.77	
Well	23	8.73 ± 5.28	
Tumor size (cm)			
<5	83	7.83 ± 3.17	0.152
*⩾*5	79	8.61 ± 3.71	

## Data Availability

The data used to support the findings of this study are included within the article.
